# Reciprocal associations between smoking cessation and depression in older smokers: findings from the English Longitudinal Study of Ageing

**DOI:** 10.1192/bjp.bp.114.153494

**Published:** 2015-09

**Authors:** Lion Shahab, Gail Gilchrist, Gareth Hagger-Johnson, Aparna Shankar, Elizabeth West, Robert West

**Affiliations:** **Lion Shahab**, PhD, Department of Epidemiology and Public Health, University College London, UK; **Gail Gilchrist**, PhD, Institute of Psychiatry, King's College London; **Gareth Hagger-Johnson**, PhD, Institute of Child Health, University College London; **Aparna Shankar**, PhD, Department of Epidemiology and Public Health, University College London; **Elizabeth West**, PhD, School of Health and Social Care, University of Greenwich, London; **Robert West**, PhD, Department of Epidemiology and Public Health, University College London, UK

## Abstract

**Background**

Depression is a particular problem in older people and it is important to know how it affects and is affected by smoking cessation.

**Aims**

To identify reciprocal, longitudinal relationships between smoking cessation and depression among older smokers.

**Method**

Across four waves, covering six years (2002–2008), changes in smoking status and depression, measured using the 8-item Centre for Epidemiologic Studies Depression Scale, were assessed among recent ex-smokers and smokers (*n* = 2375) in the English Longitudinal Study of Ageing.

**Results**

In latent growth curve analysis, smoking at baseline predicted depression caseness longitudinally and vice versa. When both processes were modelled concurrently, depression predicted continued smoking longitudinally (B(β) = 0.21 (0.27); 95% CI = 0.08–0.35) but not the other way round. This was the case irrespective of mental health history and adjusting for a range of covariates.

**Conclusions**

In older smokers, depression appears to act as an important barrier to quitting, although quitting has no long-term impact on depression.

Tobacco smoking and depressive disorders are leading contributors to the global disease burden among adults.^1,2^ Cross-sectional general population studies suggest that smokers are at least twice as likely as non-smokers to experience depression and anxiety, with smaller associations reported for older smokers.^3^ Whereas the association between smoking and mental health is well documented, showing a bidirectional relationship where uptake of smoking predisposes to poorer mental health subsequently and vice versa,^4,5^ less is known about the reciprocal impact of smoking cessation on mental health, especially among older, long-term smokers.

There are a number of reasons which have been put forward to explain the strong association between smoking and poor mental health. These include the possibility that smoking and mental health have common causes such as shared genetic vulnerability,^6^ psychosocial or environmental factors.^7^ Alternatively, those with mental health problems may either use smoking as a way to ‘self-medicate’ to regulate their emotions and symptoms or smoking may in fact exacerbate mental health problems, for instance, via its neurophysiological effects.^8^

Given these explanations, it could be speculated that stopping smoking may either improve or worsen mental health and in turn poor mental health may either undermine quit attempts or not. Research to date has not been able to provide a definitive answer. Regarding the impact of smoking cessation on mental health, some studies suggest that short- to longer-term smoking cessation leads to worse mental health, particularly among those with a history of depression.^9,10^ Conversely, smoking cessation has been linked to a decrease in stress and anxiety and no worsening in depressed mood within the first year in the general population.^11–13^ Moreover, recent systematic reviews concluded that quitting smoking does not result in an increased risk of worsening symptoms or relapse for smokers with a history of depression up to 18 months post-smoking cessation,^14^ and it may even improve long-term mental health outcomes.^15^ Regarding the impact of mental health on smoking cessation, although there is little evidence that poor mental health attenuates quit success,^16^ some studies have shown that outcomes are poorer for those with past major depressive episodes.^17^ Findings among older smokers are equally conflicting, showing both negative and positive associations of mental health problems with smoking cessation and vice versa.^18,19^

These contradictory findings may be due to several factors. Much of what is known about the impact of smoking cessation on mental health comes from randomised controlled trials rather than naturalistic, observational studies. Cohort studies that can address the issue by comparing continuing smokers with those who stop are relatively rare. Most large-scale studies in this area tend to be cross-sectional, and retrospective and prospective studies have often parochial, small samples and suffer from differential loss to follow-up. However, even large cohort studies cannot answer the question of causality regarding the association of smoking cessation with mental health owing to the problem of self-selection. For instance, it may be that smokers who stop are less prone to becoming depressed subsequently, but it is also possible that smokers who are more prone to becoming depressed upon cessation are more likely to relapse and continue to smoke. Both scenarios would be indistinguishable when looking at cohort trends (ex-smokers would look like they are improving).

The only way to differentiate these possibilities would be with a randomised controlled trial in which researchers manipulated either mental health, to observe its impact on the subsequent likelihood of quitting smoking, or smoking status, to observe its impact on subsequent changes in mental health. However, since this would be neither ethical nor practical, the current study used a prospective cohort design with parallel latent growth curve analysis to minimise the risk of self-selection bias by modelling the impact of change in smoking status on mental health and vice versa, while controlling for their interdependence. Whether or not smoking cessation has a negative impact on mental health and vice versa is of practical and clinical importance, as it will influence not only a smoker's decision to stop but also the willingness of health professionals to intervene. Given that nearly one in five among the elderly exhibit depression,^20^ we chose to investigate this issue in a cohort of older smokers and ex-smokers as this is a high-risk population and one which allows a better exploration of the long-term impact of smoking behaviour on mental health. The main research questions were: (1) What is the independent impact of smoking cessation on mental health and vice versa? (2) What is the reciprocal impact of smoking cessation and mental health on one another? (3) Does this association (if any) hold when controlling for covariates? (4) Does this association (if any) equally hold for those with and without a history of mental health problems?

## Method

### Study population and design

Data came from the English Longitudinal Study of Ageing (ELSA), a nationally representative general population panel study among those aged 50 and above. Further information can be found elsewhere.^21^ Briefly, the study sample was drawn from participants in the Health Survey for England (HSE) in 1998, 1999 and 2001 who were born before March 1952 (‘recruitment wave’). The sample is followed up every 2 years from 2002 onwards. This paper reports on data from the first four ELSA waves (Wave 1 or ‘baseline wave’ in 2002/2003, Wave 2 in 2004/2005, Wave 3 in 2006/2007 and Wave 4 in 2008/2009) of initial core members who were smokers or recent (≤1 year) ex-smokers at the recruitment wave (21.2%; 95% CI 20.4–22.0). Of these 2375 participants at Wave 1, data from 1765 (74.3%) were available at Wave 2, 1474 (62.1%) at Wave 3 and 1272 (53.6%) at Wave 4 (see Table 1).

**Table 1:** Baseline characteristics by wave

	Baseline/wave 1 (*n* = 2375)	wave 2 (*n* = 1765)	wave 3 (*n* = 1474)	wave 4 (*n* = 1272)
*Sociodemographics*				
Mean (s.d.) age	62.2 (8.8)	61.7 (8.4)***	61.1 (8.1)***	60.8 (7.9)***
% (*N*) women^a^	55.3 (1313)	55.8 (985)	57.1 (842)*	58.6 (745)**
% (*N*) White^a^	97.6 (2318)	98.1 (1732)**	98.2 (1448)*	98.5 (1253)**
% (*N*) in paid work in last month	37.6 (894)	40.1 (707)***	41.1 (606)***	43.0 (547)***
% (*N*) routine/manual occupation^a, 1^	58.6 (1368)	56.3 (977)***	55.7 (807)***	54.2 (676)***
Mean (s.d.) net wealth (in £1000)^2^	130.1(246.0)	139.5 (258.2)**	143.4 (267.1)**	152.8 (296.1)***

*Social integration*				
% (*N*) Cohabiting	64.2 (1524)	64.0 (1129)	63.2 (931)	63.8 (811)
Mean (s.d.) positive social support^3^	21.9 (7.7)	21.9 (7.7)	21.8 (7.6)	22.0 (7.4)
Mean (s.d.) close social ties^4^	5.1 (4.9)	5.0 (4.8)	5.2 (5.2)*	5.3 (5.0)*

*Health behaviours*				
% (*N*) regular alcohol drinking^5^	27.9 (661)	28.8 (508)	27.9 (410)	28.8 (366)
% (*N*) sedentary activity level^6^	8.6 (202)	6.8 (119)***	6.5 (95)***	5.8 (74)***
% (*N*) current smokers^7^	79.9 (1870)	80.5 (1407)	80.5 (1179)	79.9 (1009)
Mean (s.d.) cigarettes per day^a,8^	15.8 (9.7)	15.8 (9.7)	15.8 (9.7)	15.6 (9.7)
Mean (s.d.) age started smoking^a,8^	18.5 (9.2)	18.7 (9.6)	18.6 (9.1)	18.8 (9.9)

*Physical health*				
% (*N*) chronic illness	68.3 (1621)	66.9 (1181)*	67.4 (993)	67.1 (853)
% (*N*) ADL disabled^9^	23.7 (555)	21.4 (376)***	21.1 (310)***	21.1 (268)**
% (*N*) IADL disabled^9^	25.2 (592)	23.3 (408)***	23.5 (345)*	21.6 (274)***
% (*N*) Poor mobility^9^	49.7 (1166)	47.9 (840)**	47.7 (701)*	47.2 (598)**

*Mental health*				
Mean (s.d.) CES-D^10^	2.02 (2.2)	1.97 (2.2)	1.95 (2.2)	1.89 (2.2)**
% (*N*) depression case (CES-D)^b,10^	22.9 (525)	22.1 (381)	21.5 (311)*	20.4 (254)**
% (*N*) mental health history^a^	11.2 (267)	11.2 (198)	11.2 (165)	10.7 (136)

ADL, activities of daily living; IADL, instrumental activities of daily living; CES-D, Center for Epidemiologic Studies-Depression Scal e; GHQ, General Household Questionnaire.

a. Data come from the recruitment wave (otherwise from Wave 1/baseline wave).

b. Cut-off ≥4.

1. Data missing for 40 individuals.

2. Data missing for 24 individuals.

3. Data missing for 281 individuals.

4. Data missing for 406 individuals.

5. Data missing for 5 individuals.

6. Data missing for 32 individuals.

7. Data missing for 36 individuals.

8. Data missing for 20 individuals.

9. Data missing for 29 individuals.

10. Data missing for 86 individuals.

* Differences compared with Wave 1: *P*<0.05,

** *P*<0.01,

*** *P*<0.001.

### Measures

#### Mental health

The primary outcome, depression status, was assessed across all ELSA waves with the Centre for Epidemiologic Studies Depression Scale (CES-D),^22^ which has been validated as a reliable brief screening instrument for detecting recent symptoms of depressive disorder in elderly people in a shortened 8-item version used here.^23^ Consistent with that analysis and with a previous ELSA paper using this measure,^24^ a score of ≥4 was used to define possible/probable depression caseness. At the recruitment wave only, participants were asked about any long-standing illnesses which were coded according to the ninth revision of the International Classification of Diseases and Related Health Problems.^25^ In addition, prescription drugs were also recorded and coded according to the latest British National Formulary. Participants who either reported having mental disorders or who indicated using prescription drugs classified as hypnotics and anxiolytics, drugs used in psychoses and related disorders or antidepressants were considered to have a history of mental health problems.

#### Smoking characteristics

The other primary outcome, smoking status, was assessed at each ELSA wave by asking participants whether they had ever smoked, with those responding ‘yes’ further asked whether they smoked cigarettes at all nowadays. At the recruitment wave, the age that participants had started smoking and the number of cigarettes smoked per day were also recorded.

#### Covariates

Basic sociodemographic characteristics including age, gender, ethnicity and socioeconomic classification (routine and manual occupation/other, derived from the National Statistics Socio-Economic Classification) came from HSE in the baseline assessment. At each ELSA wave, participants were asked whether they were living with their partner and provided information on the number of family members and friends with whom they enjoyed close relationships. A self-completion questionnaire was used to derive a measure of positive social support.^26^ Participants indicated whether or not they had been in paid employment in the past month and total non-pension net wealth (divided into quintiles in analysis) was calculated from the value of any assets minus debt.^21^ Regular alcohol drinking was defined as having drunk alcohol on average on at least 3 days a week over the past 12 months. Physical activity was determined with a modified Allied Dunbar Survey of Fitness^27^ and sedentary lifestyle defined as not working or being in a sedentary occupation, engaging in mild exercise less than four times a month, with no moderate or vigorous activity. Those who indicated they had one of the following conditions were considered to suffer from a chronic illness: Alzheimer's disease, arthritis, asthma, cancer or a malignant tumour (excluding minor skin cancers), chronic lung disease, dementia, diabetes, high blood pressure/hypertension, Parkinson's disease or any emotional, nervous or psychiatric problems. Functional health was determined by questions relating to problems with mobility, activities of daily living (ADL) and instrumental ADL (IADL) as previously described.^28^

### Analysis

Group differences in continuous and categorical variables were assessed in IBM SPSS v.21 with *t*-tests and chi-square tests, respectively, and simple longitudinal associations with logistic regression analysis. To assess the independent and reciprocal longitudinal relationship between smoking and mental health trajectories across waves, we computed latent growth curve models (LGCMs, also known as ‘random effects models’) in Mplus version 6.12 using a weighted least squares estimator which is suitable for categorical outcomes and can handle missing data.^29^ LGCMs acknowledge the correlated nature of repeated measure on the same individual and make use of all available data points; it can fit latent variables (extracted from observed variables at each wave) representing intercept (predicted status at baseline) and slope (predicted change across waves) as random effects, allowed to vary between individuals. The key advantage of LGCM is that change in both outcomes can be estimated in the same model. Thus, the slope of each latent variable can be regressed on the intercept of the other simultaneously while also controlling for correlations between latent variables at baseline, their rate of change across time and correlations between baseline and rate of change for each individual latent variable. Successive models were tested, from the most restricted, simple to the least restricted (allowing all parameters to vary randomly) and complex (including all possible paths), and the most parsimonious model was chosen based on significantly improved model fit, assessed by chi-square tests. Both standardised (β) and unstandardised coefficients (*B*) are presented for latent variable associations in the model and confounding was controlled by regressing latent variable intercept and slope on relevant covariates. Given the limitations of individual fit measures, we used the Tucker–Lewis index (TLI), the comparative fit index (CFI) and the root mean square error of approximation (RMSEA) in addition to chi-square goodness of fit test to evaluate suitability of models. Both TLI and CFI values above 0.95 indicate good fit as does RMSEA below 0.06.^30^ As longitudinal sampling weights were only available for participants present at all waves, analyses were carried out unweighted to maximise use of available data. In sensitivity analysis, the sample was restricted to those with complete data to assess health survivor effects and continuous rather than categorical CES-D score was used as a measure of mental health to evaluate the robustness of findings. In a multigroup analysis, the target model was examined in participants with and without a history of mental health problems to determine whether findings applied to both groups by constraining key parameters/path coefficients to be equal across groups and exploring whether model fit (measured by chi-square goodness of fit test) significantly improved when these constraints were removed. Lastly, where appropriate, effect size or the numbers needed to treat were calculated for demonstrative and clinical purposes of the observed effect using standard methodology.^31^

## Results

Table 1 shows participant baseline characteristics as a function of follow-up across waves. Those who were lost to follow-up tended to be older, male, Black, from a manual working background, out of work, less affluent or physically fit. However, there were few other differences between those retained and lost at follow-ups, including on the main outcome measure of smoking status and mental health.

### (1) What is the independent impact of smoking cessation on mental health and vice versa?

**Fig. 1:**
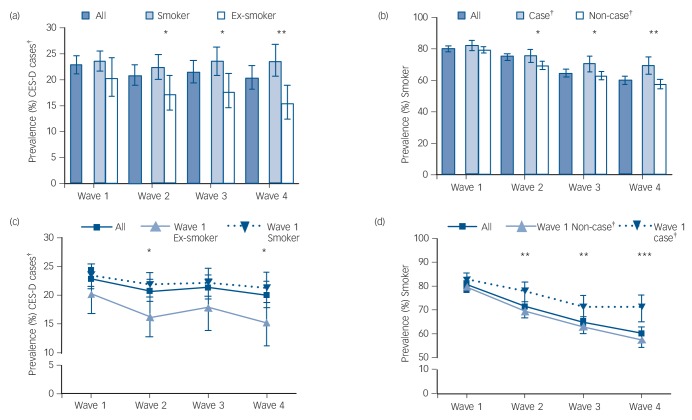
Cross-sectional prevalence of (a) CES-D caseness by smoking status and (b) smoking status by CES-D caseness and longitudinal changes in (c) CES-D caseness as a function of baseline smoking status and (d) smoking status as a function of baseline CES-D caseness. CES-D: Center for Epidemiologic Studies Depression Scale; ^†^Based on ≥4 CES-D cut-off; *P<0.05, **P<0.01, ***P<0.001.

As shown in Fig. 1, overall prevalence of CES-D caseness remained relatively constant across waves, decreasing by only a small amount, whereas smoking prevalence steadily declined. There was a negative cross-sectional and longitudinal association between CES-D caseness and smoking cessation which developed over time. Whereas at baseline (Wave 1) neither were associated with one another, in cross-sectional analysis ex-smokers at each subsequent wave were much less likely to be CES-D cases at that wave (Fig. 1a) and CES-D cases were much less likely to have quit (Fig. 1b). This was also the case when looking at longitudinal changes; those who had stopped at baseline (Wave 1) were less likely to be CES-D cases at subsequent waves (Fig. 1c) and participants who were CES-D cases at baseline (Wave 1) were less likely to have stopped smoking across follow-up (Fig. 1d). Put differently, being a smoker at baseline had a small-to-medium-sized effect (Cohen's *d* = 0.21) on becoming a CES-D case by the last follow-up and not being a CES-D case at baseline had a small-to-medium-sized effect (Cohen's *d* = 0.32) on having stopped smoking by Wave 4.

Simple LGCMs, in which the slope of the latent variable for CES-D caseness was regressed on smoking status at Wave 1 and vice versa, confirmed results: Wave 1 smoking predicted change to being a CES-D case (B (β) = 0.08 (0.16); 95% CI = 0.02–0.14) and Wave 1 CES-D caseness predicted change to being a smoker over the follow-up period (B (β) = 0.24 (0.17); 95% CI = 0.12–0.36).

### (2) What is the reciprocal impact of smoking cessation and mental health on one another?

**Fig. 2:**
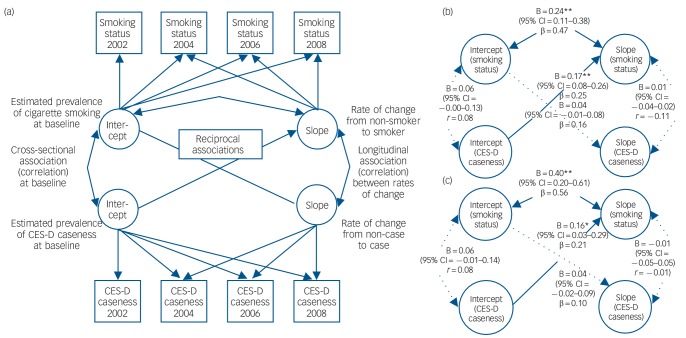
Multivariate parallel latent growth curve model: (a) conceptual model, (b) minimally adjusted model and (c) fully adjusted model. CES-D, Center for Epidemiologic Studies Depression Scale; double-headed arrows indicate correlation coefficient paths (*r*), single-headed arrows indicate probit regression coefficient paths (β); latent variables in circles, observed variables in squares; broken line indicates non-significant paths. Fit data: Model in figure part b (*n* = 2359) – RMSEA (90% CI): 0.000 (0.000–0.015); CFI: 1.000; TLI: 1.000; χ^2^(29) = 28.6, *P* = 0.486; Model in figure part c (*n* = 1900) – RMSEA: 0.010 (90% CI = 0.000–0.017), CFI: 0.999; TLI: 0.997; χ^2^(81) = 95.0, *P* = 0.137; see Table 2 for corresponding covariate coefficients of fully-adjusted model; *P<0.05, **P<0.01.

Given this independent, bidirectional association, parallel process LGCMs were used to investigate which (if any) of the two influences, smoking or mental health status, drives their relationship across waves. Successive models were tested to find the best fit to data and relationships between latent variables. As model fit was not significantly improved in the presence of a correlational path between CES-D intercept and slope, this path was not included. Figure 2a represents the final, most parsimonious conceptual model. In the model, minimally adjusted for age, gender and ethnicity, being a CES-D case across waves increased the likelihood of remaining a smoker over the follow-up period but not vice versa (see coefficients in Fig. 2b). Baseline (Wave 1) smoking (intercept) was also correlated with remaining a smoker (slope).

**Table 2:** Covariate coefficients in model fully adjusted for all baseline variables

	*B* (Standardised coefficient β)			
	Intercept (smoking status)	Intercept (CES-D caseness)	Slope (smoking status)	Slope (CES-D caseness)
*Sociodemographics*				
Age	−0.01 (−0.05)	−0.02 (−0.13)**	−0.00 (−0.05)	0.01 (0.12)
Women	0.13 (0.07)	0.26 (0.13)***	−0.10 (−0.06)	0.10 (0.15)
White	0.13 (0.02)	0.03 (0.00)	−0.53 (−0.09)*	−0.31 (−0.12)
In paid work in last month	−0.07 (−0.04)	−0.16 (−0.08)	−0.10 (−0.06)	−0.02 (−0.03)
Routine/manual occupation	−0.00 (−0.00)	0.24 (0.12)**	0.01 (0.01)	−0.06 (−0.08)
Net wealth	−0.03 (−0.05)	−0.06 (−0.08)*	−0.04 (−0.07)	−0.02 (−0.09)

*Social integration*				
Cohabiting	−0.03 (−0.01)	−0.10 (−0.05)	0.01 (0.01)	0.06 (0.09)
Positive social support	−0.01 (−0.04)	−0.03 (−0.26)***	−0.00 (−0.03)	−0.01 (−0.14)
Close social ties^4^	0.00 (0.02)	−0.00 (−0.02)	0.01 (0.05)	0.00 (0.02)

*Health behaviours*				
Regular alcohol drinking	−0.02 (−0.01)	0.04 (0.02)	0.04 (0.02)	−0.09 (−0.12)
Sedentary activity level	−0.11 (−0.03)	0.33 (0.08)**	−0.26 (−0.08)	0.08 (0.06)
Cigarettes per day^a^	0.01 (0.08)*	0.00 (0.03)	0.01 (0.13)**	0.01 (0.13)

*Physical health*				
Chronic illness	−0.25 (−0.12)**	0.22 (0.10)**	−0.08 (−0.05)	−0.01 (−0.01)
ADL disabled	−0.19 (−0.08)	0.23 (0.09)*	−0.14 (−0.08)	0.00 (0.00)
IADL disabled	0.33 (0.14)**	0.23 (0.10)*	0.11 (0.06)	0.07 (0.08)
Poor mobility	−0.10 (−0.05)	0.28 (0.14)**	−0.03 (−0.02)	0.01 (0.01)

ADL, activities of daily living; IADL, instrumental activities of daily living; CES-D, Center for Epidemiologic Studies-Depression Scale.

a. Past or present consumption depending on smoking status.

* *P*<0.05,

** *P*<0.01,

*** *P*<0.001.

### (3) Does this association hold when controlling for covariates?

The parallel process model results were also checked in a fully-adjusted model, regressing smoking status and CES-D case intercept and slope on all baseline covariates. This model provided a good fit and confirmed findings from the minimally adjusted model: CES-D caseness was associated with change in smoking status, such that participants considered to be depression cases were less likely to stop smoking (or more likely to become a smoker) across waves, whereas smoking status was not associated with changes in CES-D caseness across waves (Fig. 2c). Moreover, being a smoker at baseline (Wave 1) was correlated with a greater likelihood of remaining a smoker across waves. Other covariates evidenced expected associations. Cigarette dependence as captured by cigarette consumption was associated with a greater likelihood of being a smoker and remaining a smoker, having a chronic illness with being an ex-smoker, while poor mental health status was associated with being female, from a deprived background, inactive, lacking positive social support and having poorer health (see Table 2).

### (4) What is the reciprocal impact of smoking cessation and mental health on one another among those with history of mental health problems?

Given past evidence that vulnerable people may be particularly likely to suffer adverse mental health consequences when stopping smoking, the analysis was repeated using a multiple group model where the same conceptual model was fitted to two groups: those who at the recruitment wave were considered to have a history of mental health problems and those who were not (see Table 1). This model, minimally adjusted for age, gender and ethnicity, provided a good fit to data (RMSEA: 0.019 (90% CI = 0.010–0.027), CFI: 0.998; TLI: 0.996; χ^2^(71) = 101.5, *P* = 0.010). It confirmed the finding that being a CES-D case predicted greater likelihood of remaining a smoker across waves (B (β) = 0.18 (0.25); 95% CI = 0.08–0.28) but that smoking status did not affect changes in CES-D caseness longitudinally (B (β) = 0.03 (0.11); 95% CI = −0.02 to 0.07). Allowing path coefficients and parameter estimates to vary freely between both groups did not improve model fit (chi-square difference test χ^2^(11) = 12.7, *P* = 0.313), suggesting that overall model results applied equally well to both groups.

We conducted further sensitivity analyses to confirm findings. Complete case analysis which included only participants with data at all follow-up points yielded coefficient estimates very similar to the main analysis, indicating that a ‘healthy survivor effect’ is unlikely to have generated these findings (see online Table DS1: Model A). To ensure that results were not because of an underspecified model, we re-ran the analysis in a model which included the additional correlational path between CES-D caseness intercept and slope. Again, this did not materially alter coefficient estimates (see Table DS1: Model B). Lastly, to determine whether the observed associations were specific to CES-D caseness, the analysis was repeated using CES-D score as a continuous variable. These yielded results very similar to the main analysis (see Table DS1: Model C) but with poorer model fit indices, suggesting that data may be more suited to a categorical model.

Finally, given these findings and to quantify results, smoking status at last follow-up was regressed on cumulative exposure to CES-D caseness, controlling for age, gender and ethnicity. This analysis suggests that there is a dose–response relationship: compared with participants who did not experience depression at any wave, those who were cases at one wave (OR = 1.72, 95% CI = 1.27–2.32), two (OR = 1.73, 95% CI = 1.16–2.56), three (OR = 1.74, 95% CI = 1.04–2.91) or all four waves (OR = 3.60, 95% CI = 1.88–6.90) were progressively more likely to remain smokers at follow-up. Based on these data, this means that on average the number needed to treat (i.e. the number of smokers successfully treated for depression during the study period) to yield one additional ex-smoker at final follow-up would be 7.2 (95% CI = 5.2–12.0).

## Discussion

This study is the first to examine the reciprocal, concurrent association of smoking cessation and mental health. Confirming previous research in the general population,^4,12,32^ the results show that among older adults smoking is associated with increases in depressive symptoms longitudinally and that depression is associated with increases in smoking rates longitudinally. Although there was no association at baseline, being a smoker became progressively linked with depression and being depressed became progressively linked with continued smoking across follow-ups. Crucially, this study was able to disentangle the driving force behind the developing independent association of smoking and mental health among older people: the path of association was more pronounced from poor mental health to smoking status than from smoking status to poor mental health. After controlling for the inverse association and a range of covariates, CES-D caseness predicted the likelihood of remaining a smoker across waves but smoking status did not predict change in CES-D caseness.

Within the context of existing research, this would suggest that smoking is unlikely to be a strong causal determinant of mental health, at least in a sample of older long-term smokers. This is consistent with recent genetic analyses using Mendelian randomisation indicating that smoking is not a causal factor in depression.^33^ Moreover, given that the slopes of the latent variables did not correlate with one another suggests that changes in smoking status and depression did not occur simultaneously, as would be expected if common causes determined both. These results are contrary to studies that argue smoking cessation causes either amelioration or deterioration in mental health in older smokers.^18,19^ It implies that apparent improvements following smoking cessation may largely reflect self-selection (i.e. smokers with mental health problems are less likely to stop).

The fact that better mental health predicted change to being an ex-smoker or rather that poorer mental health was associated with continued smoking across 8 years of follow-up does not imply direct causation. Moreover, as smoking cessation is a function of both quit attempts and quit success and no information regarding quit attempts was available, this analysis is unable to differentiate whether the observed association of smoking cessation with mental health is because of a lower rate of quit attempts, a lower rate of quit success or both among those with depressive symptoms. It could be speculated that since depression and anxiety are normal withdrawal symptoms that peak within the first week following smoking cessation, typically lasting around 2–4 weeks, greater abstinence-induced short-term intensifications in pre-existing symptoms may potentially increase risk of relapse following smoking cessation in this population.^34^ In addition, consistent with the diathesis–stress model, it may be that other stressors, not captured by covariates in our analysis, may reduce the resilience of those with mental health problems and undermine quit success. Yet, the majority of studies do not find that depression results in worse smoking cessation outcomes and has a modest, if any, adverse effect on abstinence.^17^ Therefore, it may be that older smokers with mental health problems are less likely to attempt to stop smoking in the first place.

Whereas older quitters are known to experience substantial health benefits, and quit rates from different smoking cessation interventions among older smokers are comparable to those among younger smokers,^35^ their needs are often ignored; older smokers are less likely to be offered smoking cessation advice and support as health professionals perceive their smoking cessation success rates to be poor.^36^ Moreover, clinicians often fear that smoking cessation will exacerbate mental health problems in lifelong smokers, which may result in these smokers not being offered smoking cessation advice.^37^

Findings from our study do not support this belief. We find that stopping smoking does not result in a worsening of depressive symptoms among older people, irrespective of having experienced pre-existing mental health issues. This is also consistent with the only randomised controlled trial in this area, which showed that stopping smoking, if at all, is associated with improvements in mental health in the general population.^38^ A meta-analysis of 16 smoking cessation intervention trials for smokers with current or previous histories of depression reported a small, positive effect of adding behavioural mood management to smoking cessation interventions.^39^ Despite these promising results, no trials among older smokers were included. Given positive gains in survival, physical and mental health in the medium to longer term, older smokers should be supported to quit using recommended methods, whether or not they have depression. Our findings provide clinicians with the evidence that offering smoking cessation to older smokers with depression will be unlikely to worsen depressive symptoms.

### Limitations

The findings do not apply to the whole population of smokers of all ages. For instance, compared with national data, ethnic minorities are underrepresented in this sample, possibly reflecting healthy survivor effects or the fact that smokers in the UK are more likely to be White than Black in older age groups.^40^ However, by focusing on smokers aged 50 and older followed up over 8 years, the current methodology provided the opportunity to examine the mental health and smoking association with maximal effect, given participants' long-term exposure to smoking. Future studies could compare results in younger cohorts to evaluate age as a moderator of these effects. Only a single item was available to assess smoking status across waves, and we did not attempt to delineate between intermittent and continuous smokers. However, simple self-reported smoking measures are considered reliable in epidemiological studies and both measures of intensity (cigarettes per day) and chronicity (length of time of smoking) were taken into account in analysis to further differentiate among smokers. Many studies suffer from health selection at recruitment, and any longitudinal study suffers from attrition which can produce healthy survivor effects, which is particularly relevant when considering smoking and depression in an older sample as both are associated with increased mortality.^1,2^ For this reason, a wide range of relevant covariates was included in an analysis which made use of all available data. Given that findings did not differ in complete case analysis, attrition and healthy survivor effects are unlikely to have biased results. Whereas self-selection may have a role to play, the current study is the first to examine the reciprocal, concurrent association of smoking cessation and mental health using validated instruments and appropriate modelling techniques to separate self-selection from other processes.

### Clinical implications

This is the first study to examine what drives the reciprocal longitudinal association between smoking and mental health in a representative cohort of older adults. The findings show that the impact of depression on smoking status is stronger than the impact of smoking status on depression across time. Smoking cessation did not result in a worsening of depressive symptoms in the study population or a subsample with previous mental health problems. Those with depression were less likely to have stopped smoking across follow-ups. Older smokers should be encouraged to quit, whether or not they present with depression; however, additional support may be required to help those with mental health problems and treatment of mental health problems is likely to have corollary benefits in terms of physical health by increasing smoking cessation.
